# Moisturisers from birth in at‐risk infants of atopic dermatitis – a pragmatic randomised controlled trial

**DOI:** 10.1111/ajd.13703

**Published:** 2021-08-23

**Authors:** Pamela Si Min Ng, Lynette Wei Yi Wee, Valerie Pui Yoong Ho, Weixuan Colin Tan, Priya Bishnoi, Uma Alagappan, Sharon Mun Yee Wong, Emily Yiping Gan, Bin Huey Quek, Liang Shen, Bing Su, John EA Common, Mark Jean Aan Koh

**Affiliations:** ^1^ Department of Paediatric Medicine KK Women’s and Children’s Hospital Singapore; ^2^ Dermatology Service KK Women’s and Children’s Hospital Singapore; ^3^ Department of Neonatology KK Women’s and Children’s Hospital Singapore; ^4^ Saw Swee Hock School of Public Health National University of Singapore Singapore; ^5^ Research Center KK Women’s and Children’s Hospital Singapore; ^6^ Skin Research Institute of Singapore, Agency for Science, Technology and Research (A*STAR) Singapore

**Keywords:** atopic dermatitis, ceramide, filaggrin, food sensitisation, moisturiser

## Abstract

**Background:**

Atopic dermatitis (AD) is a common, chronic dermatosis, with onset of disease often manifesting in early infancy. Past studies evaluating the early use of moisturisers in the prevention of AD had mixed results.

**Objectives:**

To compare the incidence of moderate or severe AD and total incidence of AD in a cohort of ‘at‐risk’ infants treated with moisturisers from the first 2 weeks of life, to a similar group without moisturisers.

**Methods:**

We performed a single‐centre, prospective, parallel‐group, randomised study in infants with at least 2 first‐degree relatives with atopy. Subjects were randomised into either a treatment group with moisturisers or a control group without moisturisers. Participants were assessed at 2, 6, and 12 months for AD and if present, the severity was assessed using SCORAD index. We also compared the overall incidence of AD, trans‐epidermal water loss (TEWL), stratum corneum (SC) hydration, pH, and incidence of food and environmental sensitisation and allergies between both groups. Genotyping for loss‐of‐functions mutations in the *FLG* gene was conducted.

**Results:**

A total of 200 subjects were recruited, with 100 subjects in each arm. There was no significant difference in incidence of moderate or severe AD, and total incidence of AD at 12 months between the treatment and control groups. There was a lower mean SCORAD in the treatment group than in the control group, but no significant difference in TEWL, SC hydration, and skin pH. No significant side‐effects were reported.

**Conclusions:**

The early use of moisturisers in ‘at‐risk’ infants does not reduce the incidence of moderate‐to‐severe AD and overall incidence of AD in infancy.

## INTRODUCTION

Atopic dermatitis (AD) is a common, chronic, recurrent, pruritic dermatosis, affecting 20.6% of children in Singapore.^1^ Poorly controlled AD can lead to impaired quality of life.^2^, ^3^ The clinical manifestations of AD often begin in infancy, and the skin defects in AD may lead to allergic sensitisation, and other manifestations of the atopic march.^4^, ^5^ AD is associated with loss‐of‐function mutations in the *FLG* gene which encodes for profilaggrin and its derivative, filaggrin, which are important components of the stratum corneum, contributing to skin barrier integrity.^6^, ^7^ Reduced or altered ceramide levels in the epidermis also predispose to the development of AD.^8^, ^9^ It is postulated that skin barrier dysfunction can occur soon after birth in predisposed infants, preceding the development of AD.^10^ Earlier studies have shown regular application of moisturisers early in life may potentially prevent or delay the onset of AD.^11^, ^12^ However, 2 subsequent studies on the early use of moisturisers did not show similar significant results at reducing the incidence of AD.^13^, ^14^

We performed a single‐centre, prospective, parallel‐group, randomised, pragmatic study to compare the incidence of moderate or severe AD in a cohort of at‐risk infants of predominantly Asian descent, in the first year of life, with at least 2 first‐degree family members with atopy, commenced within the first 2 weeks of life on a ceramide and filaggrin‐product containing moisturiser, to a control group of infants not treated with the same moisturiser. Our hypothesis was that the early use of moisturisers in at‐risk infants will reduce the incidence of moderate or severe AD, improve skin barrier function, and reduce the incidence of AD and other atopic diseases in this cohort of children.

## METHODS

### Study population

Patients were enrolled from March 2016 to September 2018. The study was approved by the hospital’s Central Institutional Review Board. During their antenatal visits, pregnant mothers were inquired on the presence of physician‐diagnosed atopic diseases (AD, allergic rhinitis, allergic conjunctivitis, or allergic asthma) in themselves, their partners and children. Written consent was obtained from either parent prior to enrolment. Healthy term neonates of at least 37 weeks gestation with at least 2 first‐degree family members with atopy were enrolled within the first 2 weeks of life.

### Trial design and outcome measures

Participants were randomised 1:1 into treatment and control groups. Randomisation was performed using computer‐generated allocation sequence, and in balanced blocks of 8 subjects per block. Generated sequences were placed in numbered, sealed opaque envelopes opened only by study coordinators on recruitment. Participants in the treatment group were required to apply Cetaphil Restoraderm® (PRO AD Derma®) Skin Restoring Moisturizer (Galderma SA, La Tour‐de‐Peilz, Switzerland) twice daily, and bathe with Cetaphil Restoraderm® (PRO AD Derma®) Skin Restoring Wash (Galderma SA, La Tour‐de‐Peilz, Switzerland). The Cetaphil Restoraderm® (PRO AD Derma®) Skin Restoring Moisturizer is formulated with both ceramides and 2 filaggrin breakdown products: arginine and sodium pyrrolidone carboxylic acid. Caregivers in the control group were not provided with the study moisturiser or wash.

Participants were assessed for AD, according to Hanifin and Rajka criteria, at 2, 6, and 12 months by a blinded investigator, and severity of disease was scored using SCORAD. Patients with SCORAD <15 were categorised as ‘mild’ disease, and those with SCORAD ≥ 15 as ‘moderate or severe’ disease. Side effects of the study moisturiser and wash were assessed at each visit. If subjects were clinically assessed with AD at any point during the study, their randomisation allocation was revealed to only the treating physician. These subjects were treated with moisturisers, topical corticosteroids, and topical calcineurin inhibitors as deemed necessary.

At each visit, trans‐epidermal water loss (TEWL) (VapoMeter®, Delfin Technologies Ltd, Finland), stratum corneum (SC) hydration (MoistureMeterSC®, Delfin Technologies Ltd, Finland), and skin pH (CK electronic, Courage + Khazaka electronic Gmbh, Germany) were performed. For each parameter, a mean of 3 consecutive readings on both calves was recorded. At 12 months, skin prick tests (SPT) to common foods and environmental allergens were offered to all participants. Any positive SPT results were corroborated with a detailed clinical history to determine sensitisation versus true food allergy.

Participants were also assayed for loss‐of‐function (LoF) mutations in the *FLG* gene. Briefly, DNA was extracted and purified from de‐identified buccal mucosa swabs collected with the Oragene DNA OG‐500 kit (DNA Genotek) and screened for *FLG* mutations at the Skin Research Institute of Singapore, Agency for Science, Technology and Research (A*STAR). Whole gene sequencing and analysis was completed using a multiplexed microfluidic PCR assay coupled with Illumina next‐generation sequencing and an established bioinformatics pipeline.^15^

Our primary outcome was to evaluate the difference in incidence of moderate or severe AD in at‐risk infants treated with moisturisers within the first 2 weeks of life, compared to those without moisturisers. Secondary outcome measures were overall incidence of AD, TEWL, SC hydration, skin pH, incidence of food and environmental sensitisation and allergies, and loss‐of‐function mutations in the *FLG* gene. The side‐effect profiles of the prescribed moisturisers and wash were also assessed.

### Power calculation and statistical analyses

Power calculation was performed using software Strata 12. To achieve a power of 80% and alpha of 0.05, and to detect a difference of 20% in the incidence of moderate or severe AD between the treatment and control groups, the number needed was calculated to be 91 in each group. To account for an estimated dropout rate of 10%, we recruited 100 subjects in each group. Statistical analysis was performed using software SPSS version 26. Data were analysed according to intention‐to‐treat analysis. Descriptive analysis was performed for demographic and baseline characteristics. Mean (SD) or median (range) was used for numerical variables, and N (percentage) was used for categorical variables. Independent samples t‐test was used to compare the incidence rate of moderate or severe AD, TEWL, SC hydration and skin pH at each visit, between both groups. Incidence of overall AD, food sensitisation, or allergies were compared using chi‐square test or Fisher exact test.

## RESULTS

### Patients

**Figure 1:**
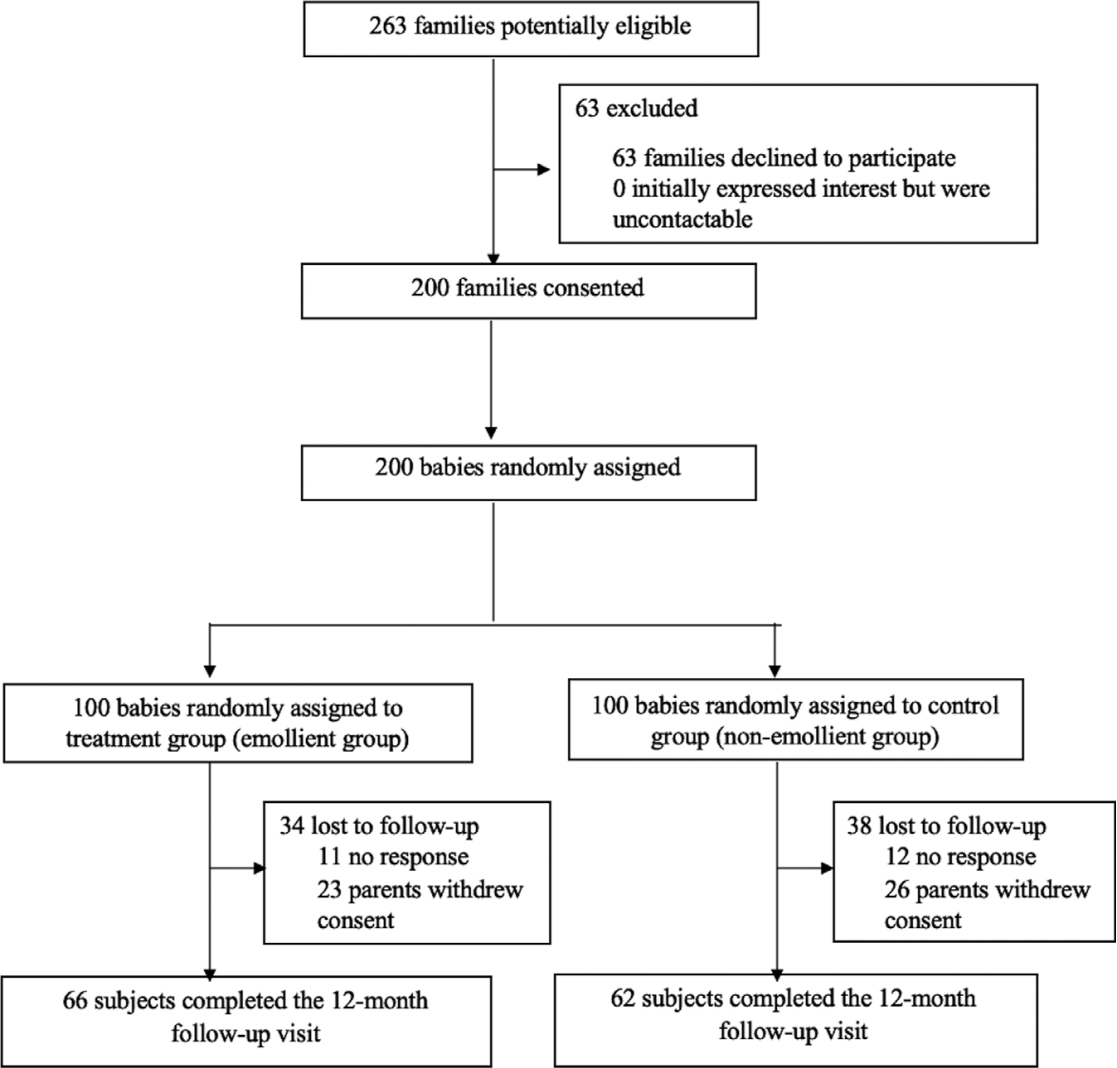
Study flow diagram.

A total of 200 subjects were recruited, with 100 subjects in each group. Except for SC hydration, there was no significant difference in baseline characteristics between the groups (Table 1). Total dropout rate was 36.0%, with marginally higher rate in the control group compared to the treatment group (38% vs. 34%) (Figure 1).

**Table 1:** Baseline characteristics of subjects in treatment and control groups

	Treatment group (*n* = 100)	Control group (*n *= 100)	*P*‐value
Gender			
Male	52	47	0.479
Female	48	53	
Race			
Chinese	56	51	0.078
Malay	32	32	
Indian	4	14	
Others	8	3	
Birth weight, mean ± standard deviation (kg)	3.08 ± 0.43	3.06 ± 0.38	0.697
Maternal antenatal conditions, *n* (%)^†^	26 (26.0%)	18 (18.0%)	
Gestational diabetes	19	10	0.071
Pre‐eclampsia/ Pregnancy‐induced hypertension	1	4	0.174
Smoking	5	4	0.733
Others^‡^	4	3	0.700
Baseline skin pH, mean ± standard deviation	6.61 ± 0.95	6.40 ± 0.74	0.324
Baseline trans‐epidermal water loss (TEWL), mean ± standard deviation	7.51 ± 3.29	7.48 ± 2.93	0.946
Baseline stratum corneum (SC) hydration, mean ± standard deviation	13.74 ± 4.40	12.38 ± 2.87	0.02

^†^ Certain subjects have multiple conditions.

^‡^ Other maternal antenatal conditions include asthma, bipolar disorder, hyperthyroidism.

### Primary outcome

The incidence of moderate or severe AD was 5.0% in the entire cohort. This constituted 13.9% of all subjects who developed AD. There was no significant difference in incidence between the treatment group (4.0%) and the control group (6%) (*P* = 0.603).

### Secondary outcomes

**Figure 2:**
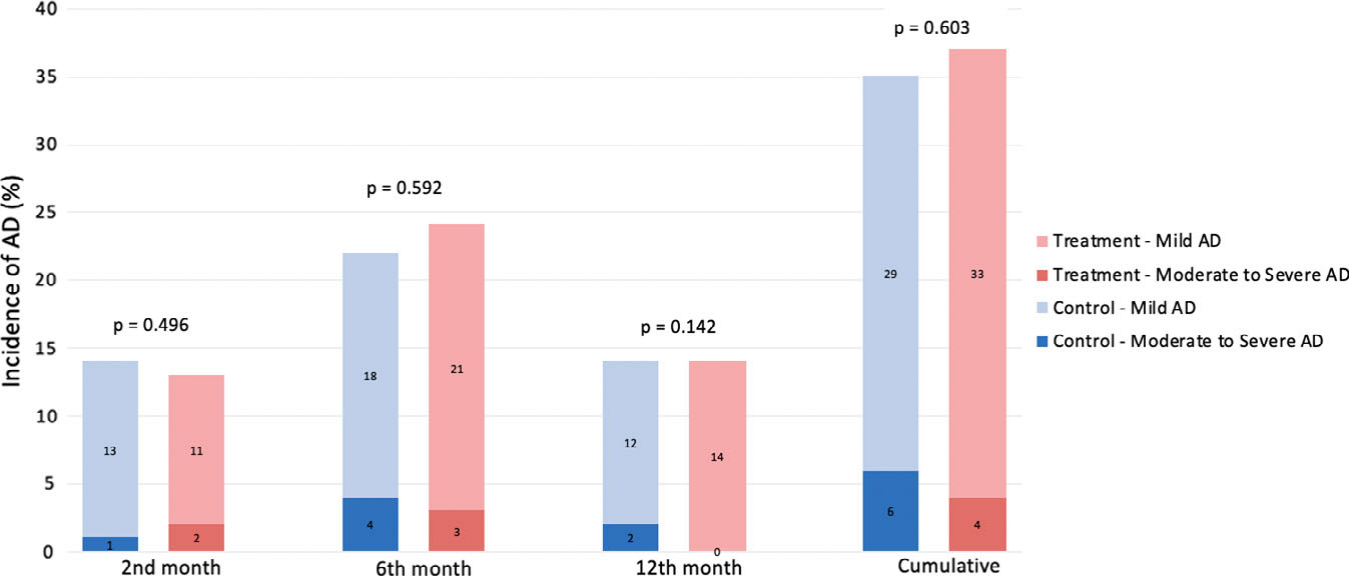
Total incidence of moderate and severe AD at each study visit between treatment and control groups.

**Figure 3:**
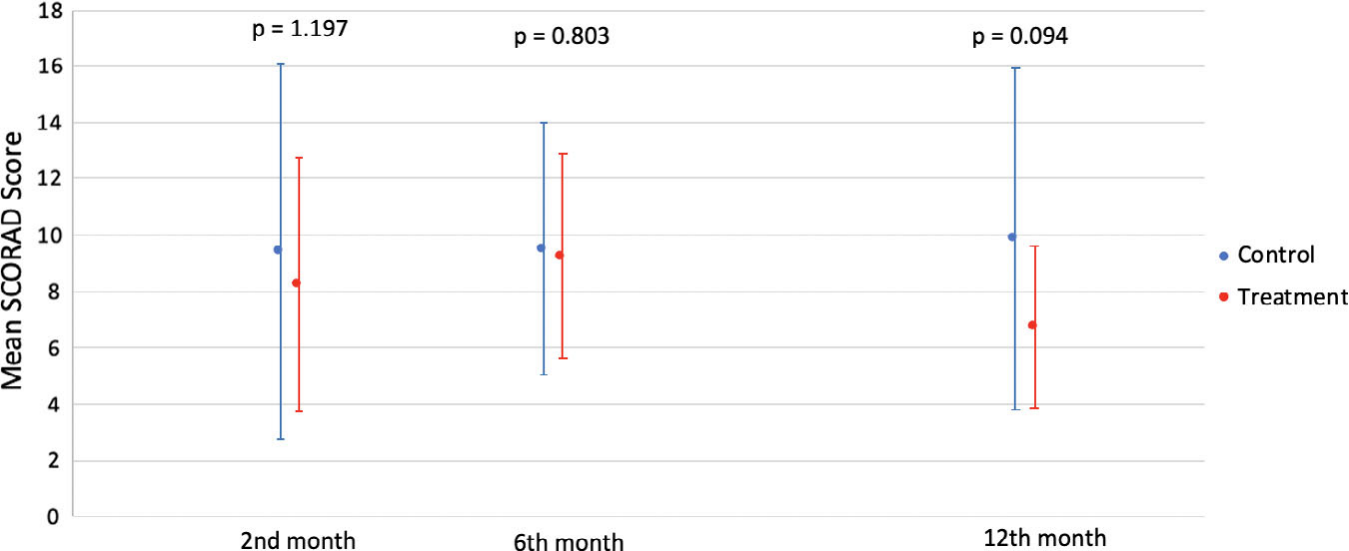
Mean SCORAD score over time in treatment and control groups.

The overall incidence of AD in our cohort was 36.0%, with no significant difference between treatment (37.0%) and control (35.0%) groups (*P *= 0.768) (Figure 2). The mean SCORAD was lower in the treatment group at each visit, but this was not statistically significant (Figure 3). There was no significant difference with regard to age of diagnosis of AD in both groups, with 13, 18, and 6 patients in the treatment group, and 14, 14, and 7 patients in the control group being diagnosed at the 2‐, 6‐, and 12‐month time points in the study.

**Figure 4:**
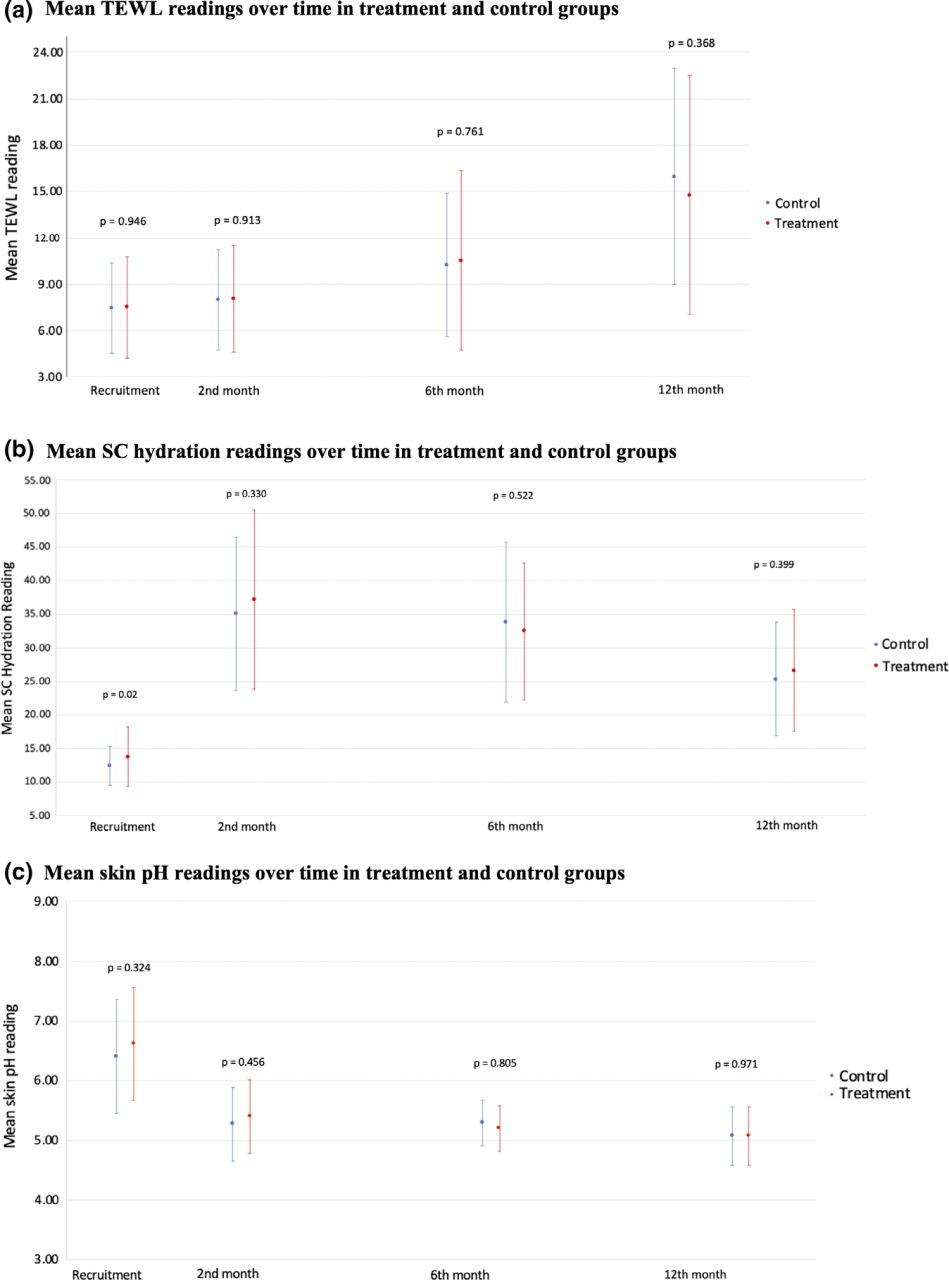
(a) Mean TEWL readings over time in treatment and control groups. (b) Mean SC hydration readings over time in treatment and control groups. (c) Mean skin pH readings over time in treatment and control groups.

There was a trend for increasing TEWL over time, but no significant difference between both groups at all 3 time points (Figure 4a). There was a general increase in SC hydration from enrolment to 2 months, with subsequent decrease from 6 to 12 months (Figure 4b), and decreasing skin pH over time (Figure 4c), but these were not statistically significant.

**Figure 5:**
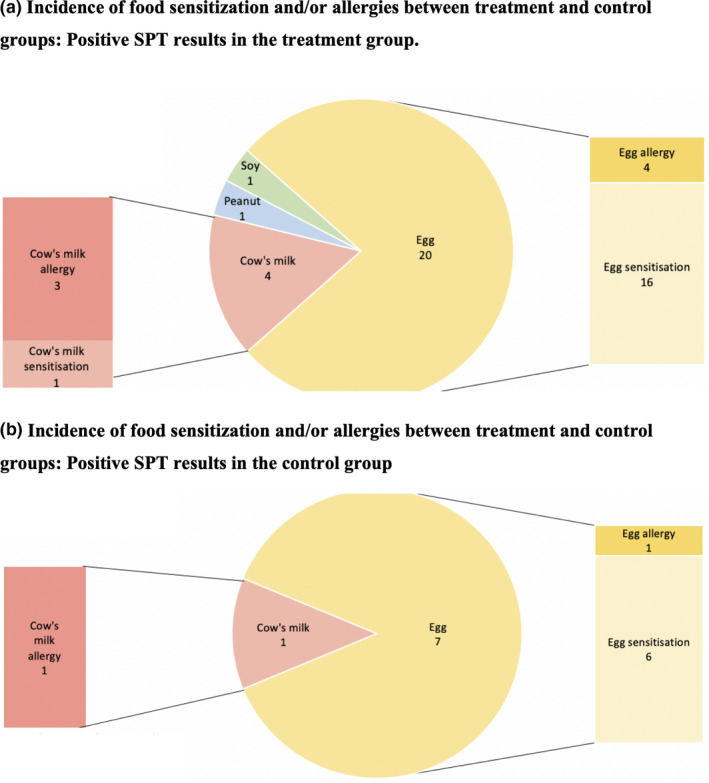
Incidence of food sensitisation and/or allergies between treatment and control groups. (a) Positive SPT results in the treatment group. (b) Positive SPT results in the control group.

Seventy‐three subjects underwent SPT (38 in treatment group, 35 in control group) with 39.7% of these with at least 1 positive result (Figure 5). There was a significantly higher proportion of subjects in the treatment group with at least 1 positive result (55.3%) compared to the control group (22.9%) (*P *= 0.005). The commonest positive SPT was to eggs (52.6% in treatment group, 20.0% in control group) but only 5 of the 27 positive subjects (4 in treatment group, 1 in control group) reported clinically relevant allergic reactions. Of these 5 subjects, 4 had been diagnosed with AD. There were 4 subjects in the treatment group (4%) and 1 subject in the control group (1%) with positive SPT to cow’s milk, of which 3 in treatment group and the 1 in control group developed allergic reactions. Six subjects developed positive reaction to house dust mite (HDM), with 4 in treatment and 2 in control group.

*FLG* gene testing was done in 131 participants, of which 8 returned positive for one or more *FLG* LoF mutations (3 in the control group, 5 in the treatment group). The *FLG* mutations detected were p. Arg3733X, p.K4022X, c.3321delA, c.3758delG, and c.3222del4. Four participants had the p.K4022X mutation, and 3 participants had the p. Arg3733X, c.3758delG, and c.3222del4 mutations respectively. One participant in the treatment group with mild AD had both p.K4022X and c.3321delA mutations. Of the 8 participants with *FLG* mutations, 6 (2 in the control group and 4 in the treatment group) developed AD during the 12‐month follow‐up period, of which only 1 patient with a c.3222del4 mutation developed moderate‐to‐severe AD.

### Safety and side effects

The use of Cetaphil Restoraderm® (PRO AD Derma®) Skin Restoring Moisturizer and wash in our cohort of infants was generally safe. Only 1 patient from the treatment group developed an irritant contact dermatitis on the back of his ears during the 2‐month review. No other side effects were reported throughout the study.

## DISCUSSION

An early study by Simpson *et al*. in 2010 showed promising results of the early use of moisturisers in infants at risk of developing AD.^16^ Twenty‐two neonates at high risk for developing AD used emollient therapy from birth. No intervention‐related adverse events were reported and only 15.0% developed AD, suggesting a protective effect when compared with historical controls. Two further studies in 2014 also showed promising results. Horimukai *et al*. recruited 118 neonates at high risk of developing AD. An emulsion‐type moisturiser was applied daily during the first 32 weeks of life to half the subjects, and 32% fewer neonates in the treatment group developed AD by week 32 compared to the control group.^12^ Simpson *et al*. recruited 124 neonates at high risk for AD, with subjects in the intervention arm treated with emollients at least once per day. The study found a statistically significant reduction in the cumulative incidence of AD with a relative risk reduction of 50%.^11^

However, further research has not shown similar encouraging results. McClanahan *et al*. enrolled infants at high risk for developing AD, with treatment group receiving once‐daily application of a similar moisturiser as our current study, and the control arm was not provided with the same moisturiser but could use an emollient of their choice. This study enrolled 54 subjects in the treatment group and 46 subjects in the control group. Across all outcomes, there was a trend in favour of the intervention group, without reaching statistical significance. AD was diagnosed in 13.2% vs. 25.0% at 12 months, and 19.4% vs. 31.0% at 2 years in intervention vs. control groups, respectively. This study also found no significant differences in skin barrier or microbiome assessments between the intervention and control groups.^13^ The largest study investigating the early use of moisturisers in at‐risk infants was the BEEP trial, a large, multi‐centre, pragmatic, parallel‐group, randomised controlled trial in the UK. A total of 1,394 newborns were recruited, with 693 subjects assigned to the treatment group with daily application of an emollient (Diprobase cream or DoubleBase gel), and 701 subjects assigned to the control group where only standard skin‐care advice was provided. At 2 years, there was no difference in the incidence of AD in both groups. However, there was an increased risk of skin infections seen in the emollient group compared to the control group. The authors concluded that they found no evidence that early use of daily emollient therapy prevented eczema in high‐risk children, and there was some evidence to suggest an increased risk of skin infections.^14^

Our study found an overall incidence of moderate or severe AD of 5.0% in our entire cohort, with no significant difference between the treatment and control groups. The overall incidence of AD was 36.0%, with similar incidence in both the treatment and control arms. Although these results corroborate the findings of the later studies, we postulate that possible reasons why we did not identify a difference in incidence between the two groups are because of the lack of statistical power and the pragmatic nature of the study, with a significant proportion (35%) of caregivers in the control group using over‐the‐counter moisturisers in their infants. In addition, there was a significant number of dropouts (36.0%) from the study, mostly prior to the 2‐month review. We postulate that these patients may have no or mild AD, and whose parents may have decided not to return for further evaluation. Interestingly, we found higher rates of food sensitisation in our treatment group (55.3%) compared to our control group (22.9%). However, actual food allergy was only conclusively proven in 31.0% of all patients who underwent SPT, with most of these subjects (72.4%) having already developed AD. Horimukai *et al*. also showed no statistically significant effect of emollient use on allergic sensitisation.^12^

Several case–control cohort studies have suggested that *FLG*‐null mutations constitute the strongest risk factors for AD. Chen *et al*. reported a strong association of *FLG*‐null mutations in Singaporean Chinese patients with childhood and adolescent AD. Of this cohort, 5.9% had mild, 54.6% had moderate, and 39.5% had severe AD.^17^ In our study, only 8 of the 131 participants tested had *FLG* LoF mutations, with 6 of them developing AD and 1 with moderate‐to‐severe AD. Given the small numbers, it was difficult to ascertain if the outcome of early use of emollients could have been affected by the *FLG* status. McClanahan *et al*. also performed *FLG* analysis by testing every infant for *FLG* gene mutations via buccal saliva samples at the 6 months visit, of which only 5 of the participants had a *FLG* mutation, and they were similarly unable to conclude if the early emollient use was modified by *FLG* status.^13^ In the BEEP trial, approximately 15% of their participants in the treatment and control group had one *FLG* mutation, whereas <1% had two *FLG* mutations. However, they only tested the most common *FLG* null mutations in white European populations (2282del4, R501X, S3247X, and R2447X), which were not found in our study population.^17^

With regard to safety, only 1 subject in our study developed an irritant contact dermatitis to the study moisturiser, with no other side effects reported in the other subjects. Compared to the BEEP study, none of our subjects developed skin infections.^14^

In conclusion, our study did not demonstrate a difference in the overall incidence of AD or the incidence of moderate or severe AD in the treatment and control groups, and the early use of moisturisers in at‐risk infants does not appear to reduce the risk of developing AD.

## ETHICAL APPROVAL

The study was approved by the hospital’s Central Institutional Review Board. Trials in Singapore are not required to be registered.
